# Synergistic induction of CCL2/MCP-1 expression driven by oncostatin M and IL-1*β* in human proximal tubular cells depends on STAT3 and p65 NF*κ*B/RelA

**DOI:** 10.14814/phy2.12298

**Published:** 2015-02-23

**Authors:** Rita Sarközi, Ulrike Corazza, Jan-Philipp Osterkamp, Markus Pirklbauer, Gert Mayer, Herbert Schramek

**Affiliations:** Department of Internal Medicine IV, Nephrology and Hypertension, Medical University of InnsbruckInnsbruck, Austria

**Keywords:** ccl2, oncostatin M, proximal tubular cell

## Abstract

In response to tubular injury, production, and secretion of cytokines, chemokines or extracellular matrix components by human proximal tubular epithelial cells (PTC) directly contribute to the development of tubulointerstitial inflammation and fibrosis. Here, we report a novel stimulatory and synergistic effect of oncostatin M (OSM) on proinflammatory CCL2/MCP-1 mRNA expression in human PTC. Although OSM inhibited IL-1*β*- and TNF-*α*-mediated mRNA expression of matricellular proteins TSP-1 and tenascin C (TNC), it acted synergistically with these two proinflammatory cytokines to induce CCL2 mRNA expression for up to 24 h. Stimulation of two independent human PTC lines with OSM alone led to a rapid and strong induction of this chemokine within the first hour of ligand administration, which subsequently returned toward basal levels in between 3 and 24 h and finally switched into a significant OSM-mediated 70% inhibition of basal CCL2 mRNA expression after 48 h of incubation. In contrast to OSM, which stimulated both STAT1/3 and ERK1/2 signaling, IL-1*β* led to a strong phosphorylation of p65 NF*κ*B/RelA, SMAD2/3, and p38 MAPK in human PTC. Selective silencing of these signaling molecules revealed that p65 NF*κ*B/RelA is involved in IL-1*β*-mediated stimulation of CCL2 mRNA, and that superinduction of CCL2 mRNA expression in the presence of both OSM and IL-1*β* at least partially depends on STAT3 signaling. Thus, with respect to the expression of the proinflammatory chemokine CCL2, OSM may stimulate acute inflammation via its synergistic effect with other proinflammatory cytokines early after injury.

## Introduction

ONCOSTATIN M (OSM) REPRESENTS a cytokine, which has a role in physiological and pathophysiological mechanisms such as inflammation, remodeling of extracellular matrix, hematopoiesis, and modulation of cell growth and differentiation (Tanaka and Miyajima 2003; Richards 2013). Cell types that express OSM include activated macrophages, neutrophils, monocytes, T cells, and dendritic cells (Tanaka and Miyajima 2003; Richards 2013). However, OSM also exerts distinct biological activities on a variety of cells in vivo and in vitro including the stimulation of cytokine release from cells during inflammation (Tanaka and Miyajima 2003). In kidneys, inflammatory cell invasion would not occur without signals being generated and sent out from renal parenchymal cells such as tubular epithelial cells, dendritic cells, pericytes, and endothelial cells (Molitoris 2014). These renal cell types are responsible for sensing and mediating initial signals in response to injury. To transduce its signals in human, OSM binds gp130 with low affinity and as such has little to no biological activity unless a second receptor chain is recruited, either the leukemia inhibitor factor (LIF) receptor *α* (LIFR*α*) or the specific OSM receptor *β* chain (OSMR*β*) (Gearing et al. 1992; Mosley et al. 1996; Tanaka and Miyajima 2003).

Despite the original identification and cloning of human OSM in between 1986 and 1989, there is still a considerable amount that is not clear about its biology and function in vivo (Tanaka and Miyajima 2003). While several studies have explored OSMs role in conditions with chronic inflammation including rheumatoid arthritis, lung and skin inflammatory disease (Richards 2013), only a few studies have focused on its function in renal inflammation and disease progression. OSMR*β* expression was reported to be upregulated after exposure of kidney epithelial cells to activated peripheral blood mononuclear cell-conditioned medium, which contained high levels of OSM and promoted alterations in tubular epithelial cell differentiation (Nightingale et al. 2004). In a different study performed in human proximal tubular cells (PTC), OSM attenuated the expression of epithelial marker proteins and increased the expression of mesenchymal markers (Pollack et al. 2007). These effects were associated with OSM-induced human PTC scattering in three-dimensional collagen matrices after long-term incubation, all together suggesting that OSM is able to induce cellular events indicative of tubular epithelial-mesenchymal transition (EMT) (Pollack et al. 2007). Although unilateral ureteral obstruction (UUO), a model of renal fibrogenesis, increased OSM and OSMR expression in a time-dependent manner (Elbjeirami et al. 2010), we obtained evidence from preliminary microarray analysis of OSM-stimulated human PTC that this IL-6 family cytokine might also have antifibrotic effects (Pollack et al. 2007). Indeed, we were able to show that OSM represents an inhibitor of TGF-*β*1-induced matricellular protein expression, namely of CTGF, SPARC, tenascin C (TNC) and thrombospondin-1 (TSP-1) (Sarközi et al. 2011). Its inhibitory effect on TGF-*β*1-induced CTGF mRNA expression started after 2 h of cytokine administration and lasted for at least for 24 h. In addition, when OSM was administered as a single ligand, it exerted a time-dependent dual effect on CTGF mRNA expression in human proximal tubular HK-2 cells (Sarközi et al. 2012). At early time points (between 15 min and 1 h of ligand administration) this cytokine led to a robust but transient induction of CTGF mRNA expression followed by a strong and long-lasting inhibition of basal and TGF-*β*1-mediated upregulation of CTGF mRNA, which was mainly driven by STAT3 (Sarközi et al. 2012).

Besides its possible role in tubulointerstitial fibrogenesis recent studies provided evidence for a function of OSM in the renal inflammatory response, which seems to be an important pathway for development and progression of diabetic nephropathy and other renal pathologies (Tang and Lai 2012; Kanasaki et al. 2013). Microarray experiments utilizing human renal transplant biopsies, for example, revealed an intrinsic renal acute phase response, which was associated with a significant upregulation of OSMR*β* suggesting that OSM/OSMR signaling may be of pathophysiological relevance (Mueller et al. 2008). This idea was corroborated by experiments performed in human PTC, where OSM resulted in a significantly altered expression of acute phase genes when compared with IL-6 or LIF (Luyckx et al. 2009). Additional data showing proinflammatory roles of OSM in joint, skin, lung, and vascular diseases suggest that OSM's effects in vivo depend on the context of the particular tissue/organ and the cytokine milieu present (Richards 2013).

The exact participation of OSM in renal inflammatory disease processes and the underlying cellular mechanisms along the proximal tubule are still poorly understood, partially due to its complex interplay with other cytokines. Thus, we aimed at investigating potential pro- or anti-inflammatory effects mediated by OSM in human PTC. Specifically, we studied OSM's effects on the regulation of the proinflammatory chemokine C–C motif ligand 2 (CCL2; monocyte chemoattractant protein 1; MCP-1) expression both in the absence and in the presence of proinflammatory ligands such as interleukin-1*β* (IL-1*β*) or tumor necrosis factor-*α* (TNF-*α*).

## Methods

### Reagents

Cell culture reagents were obtained from Gibco (Life Technologies, Lofer, Austria). OSM was purchased from Sigma (St. Louis, MO, USA), IL-1*β* was obtained from R&D Systems (Minneapolis, MN, USA), TGF-*β*1 and TNF-*α* were purchased from PeproTech (PeproTech, Vienna, Austria). All other reagents were obtained from Sigma.

### Cell culture

Human kidney 2 (HK-2) cells were cultured in Keratinocyte–Serum-Free Medium (KSFM) containing 10% fetal bovine serum, 5 ng/mL recombinant epidermal growth factor (rEGF), 0.05 mg/mL bovine pituitary extract (BPE), 100 units/mL penicillin, and 100 *μ*g/mL streptomycin (Ryan et al. 1994; Pollack et al. 2007; Sarközi et al. 2011, 2012). Cells were grown at 37°C in a humidified 5% CO_2_ atmosphere, and split at a 1:5 ratio, once a week. After growth to subconfluent state, cells were washed once, made quiescent by incubation in serum- and supplement-free medium for 48 h, and then used for experiments. Stimulations with OSM, TGF-*β*1, TNF-*α,* or IL-1*β* were performed in the absence of serum and any other growth supplements. RPTEC/TERT1 cells were propagated in DMEM-Ham's F-12 5 mmol/L glucose (1:1) medium supplemented with 5 *μ*g/mL insulin, 5 *μ*g/mL transferrin, 5 ng/mL sodium selenite, 10 ng/mL recombinant human EGF, 36 ng/mL hydrocortisone, 2 mmol/L l-glutamine, 100 units/mL penicillin and 100 *μ*g/mL streptomycin (Wieser et al. 2008; Sarközi et al. 2011). Cells were grown at 37°C in a humidified 5% CO_2_ atmosphere, and split at a 1:3 ratio once a week or cultured to confluence, serum- and supplement-starved for 48 h, and then used for experiments.

### siRNA-mediated gene silencing

HK-2 cells were cultured under serum- and supplement-free conditions at 60–70% confluency and transfected with ERK1, ERK2, NF*κ*B1, p65 NF*κ*B/RelA, SMAD2, SMAD3, STAT1, or STAT3 ON-TARGETplus SMART pool siRNA constructs (Thermo Scientific, Lafayette, CO, USA) or negative control siRNA (ON-TARGETplus nontargeting pool siRNA; Thermo Scientific) at a concentration of 50 nmol/L utilizing DharmaFect 1 Transfection Reagent (Thermo Scientific) according to the manufacturer's instructions. Seventy-two hours posttransfection cells were left untreated or stimulated with 10 ng/mL OSM alone, 10 ng/mL IL-1*β* alone or with a combination of the two cytokines and utilized for real-time PCR analysis.

### RNA isolation and real-time PCR analysis

Total RNA was isolated by utilizing RNeasy® Mini Kit (Qiagen, Valencia, CA, USA). For each sample 1 *μ*g RNA was reverse transcribed into cDNA with the High Capacity cDNA reverse Transcription kit (Applied Biosystems, Foster City, CA, USA) according to the manufacturer's instructions. The cDNA was analyzed on the 7500 Fast Real-Time PCR System (Applied Biosystems) using the following TaqMan® Gene Expression Assays: GAPDH (Hs99999905_m1), CCL2/MCP-1 (Hs00234140_m1), ERK1 (Hs00946872_m1), ERK2 (Hs01046830_m1), NF*κ*B1 (Hs00765730_m1), p65 NF*κ*B/RelA (Hs00153294_m1), SMAD2 (00183425_m1), SMAD3 (Hs00969210_m1), STAT1 (Hs01013996_m1), STAT3 (Hs01047580_m1), TNC (Hs00233648_m1), TSP-1 (Hs00962914_m1). Reactions were prepared in duplicate for each sample and incubated at 50°C for 2 min, 95°C for 10 min followed by 40 cycles of 95°C for 15 sec and 60°C for 1 min. A relative quantification method was used to analyze the real-time PCR data. The fractional cycle number at which the amount of amplified target reached a threshold is the CT number, the mean CT for each gene was determined and the relative amounts of transcripts for each gene was normalized to the reference gene GAPDH as follows: ΔCT = CT (gene of interest) − CT (GAPDH). The ΔCT was linearized according to the formula 2^−ΔCT^ to determine the relative expression of each gene of interest.

### Western blot analysis

Cells were washed with ice-cold phosphate-buffered saline (PBS) and lysed in RIPA lysis buffer as previously described (Pollack et al. 2007; Sarközi et al. 2011, 2012). The protein content of the samples was determined using a microbicinchoninic acid assay (Pierce, Fisher Scientific, Vienna, Austria). Cell lysates were matched for protein, separated on 10% SDS-PAGE, and transferred to a polyvinylidene difluoride microporous membrane. Subsequently, membranes were probed with one of the following antibodies: SMAD2 (L16D3), P-SMAD2 (Ser465/467), SMAD3 (C67H9), P-SMAD3 (Ser423/425), P-STAT1 (Tyr701), P-STAT3 (Tyr705), P-p38 MAPK, P-p65 NF*κ*B/RelA (Cell Signaling Technology, Danvers, MA, USA), ERK2 (C-14), p65 NF*κ*B/RelA, and P-SMAD2/3 (Santa Cruz Biotechnology, Santa Cruz, CA, USA). After extensive washing of the sheets in TBS, 0.1% Tween-20, the primary antibodies were detected using horseradish peroxidase conjugated goat anti-rabbit IgG or rabbit anti-goat IgG (Santa Cruz Biotechnology) and visualized by LumiGLO Western Blot Detection system (Cell Signaling Technology).

### Statistical analysis of quantitative real-time PCR

All values are expressed as mean ± SEM. Comparison of two groups was made by *t*-test. *P* values < 0.05 were considered significant.

## Results

### Inhibitory effect of OSM on IL-1β- and TNF-α-induced mRNA expression of TSP-1 in the human PTC line HK-2

**Figure 1:**
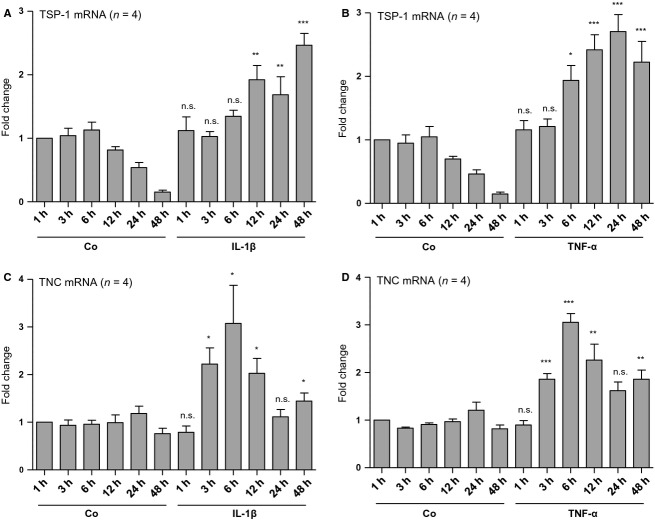
Time-dependent effects of IL-1*β* and TNF-*α* on mRNA expression of TSP-1 and tenascin C (TNC) in human proximal tubular epithelial cells (PTCs). HK-2 cells were serum- and supplement-starved for 48 h and subsequently stimulated with 10 ng/mL IL-*β*1 (A, C) or 10 ng/mL TNF-*α* (B, D) for the indicated periods of time when compared with unstimulated controls. Real-time PCR analysis was performed as described in Methods. Data are given as fold induction above TSP-1 (A, B) or TNC (C, D) mRNA control levels after normalizing to GAPDH mRNA expression. Each data point represents the average of four independent experiments with error bars corresponding to SEM (*P <* *0.05; **P <* *0.01; ***P <* *0.001).

**Figure 2:**
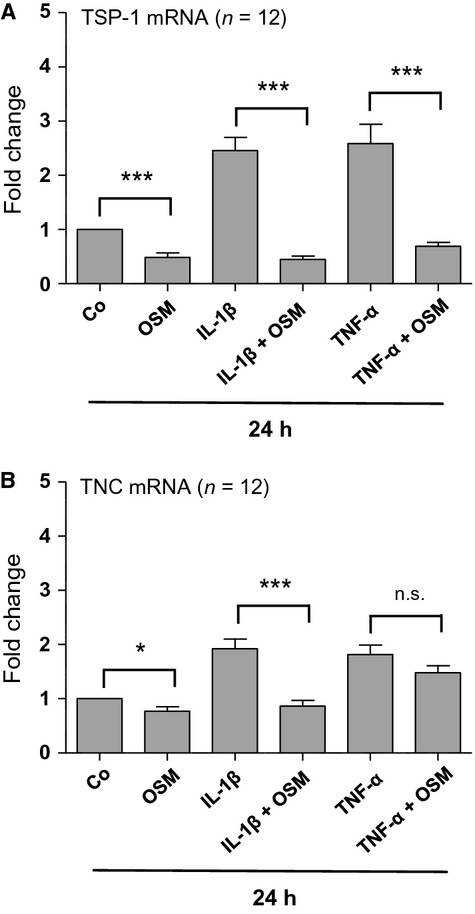
Effects of oncostatin M (OSM) on TSP-1 and tenascin C (TNC) mRNA expression in the absence and in the presence of IL-1*β* or TNF-*α*. HK-2 cells were serum- and supplement-starved for 48 h, and were then stimulated for 24 h with OSM alone, IL-1*β* alone, TNF-*α* alone (at a concentration of 10 ng/mL each) or with OSM in the presence of either IL-1*β* (IL-1*β* + OSM) or TNF-*α* (TNF-*α* + OSM). Real-time PCR analysis was performed as described in Methods. Data are given as fold induction above TSP-1 (A) or TNC (B) mRNA control levels after normalizing to GAPDH mRNA expression. Each data point represents the average of 12 independent experiments with error bars corresponding to SEM (*P <* *0.05; ***P <* *0.001).

Matricellular proteins such as TSP-1, TNC, connective tissue growth factor (CTGF), or secreted protein acidic and rich in cysteine (SPARC) show low levels of basal expression in healthy adult tissue but prompt upregulation in response to injury, during wound healing and tissue remodeling (Bornstein 2009; Chiodoni et al. 2010). In kidneys, increasing evidence exists that several of these matricellular proteins are involved in the development of tubulointerstitial fibrogenesis and renal disease progression (Sarközi et al. 2011). As we recently found that OSM represents a potent inhibitor of TGF-*β*1-induced matricellular protein expression in human PTC (Sarközi et al. 2011), we now studied its potential effects on TSP-1 and TNC mRNA expression after stimulation with proinflammatory mediators IL-1*β* and TNF-*α*. 10 ng/mL of either IL-1*β* or TNF-*α* led to a time-dependent upregulation of both TSP-1 and TNC mRNA expression in HK-2 cells, which lasted for at least 48 h (Fig.1). Highest TSP-1 mRNA expression was 2.5- and 2.7-fold after IL-1*β* or TNF-*α* administration, respectively (*n* = 4; *P* < 0.001) (Fig.1A,B), while maximum induction of TNC mRNA was 3.1-fold using either one of these two ligands (*n* = 4; *P* < 0.01 and *P* < 0.05) (Fig.1C,D). Thus, both IL-1*β* and TNF-*α* led to a long-lasting stimulation of the profibrotic genes TSP-1 and TNC, albeit lower in size when compared to those values obtained and published for TGF-*β*1 (Sarközi et al. 2011). Following 24 h of coincubation, 10 ng/mL OSM inhibited TSP-1 mRNA expression induced by both IL-1*β* or TNF-*α* (*n* = 12; *P* < 0.001) (Fig.2A). In contrast, OSM significantly inhibited TNC mRNA expression only in IL-1*β*- but not in TNF-*α*-stimulated HK-2 cells (*n* = 12; *P* < 0.001) (Fig.2B). When administered alone, both IL-1*β* and TNF-*α* led to a significant stimulation of TSP-1 and TNC mRNA expression. Furthermore, OSM alone significantly reduced basal mRNA levels of both TSP-1 and TNC after 24 h of incubation (*n* = 12; *P* < 0.001 and *P* < 0.05) (Fig.2). These results suggest that OSM has a similar inhibitory effect on TSP-1 and TNC expression induced by the proinflammatory mediators IL-1*β* or TNF-*α* as we have observed for TGF-*β*1-mediated matricellular protein expression (Sarközi et al. 2011).

### Time-dependent effects of IL-1β, TNF-α, TGF-β1, and OSM on CCL2/MCP-1 mRNA expression in human PTCs

**Figure 3:**
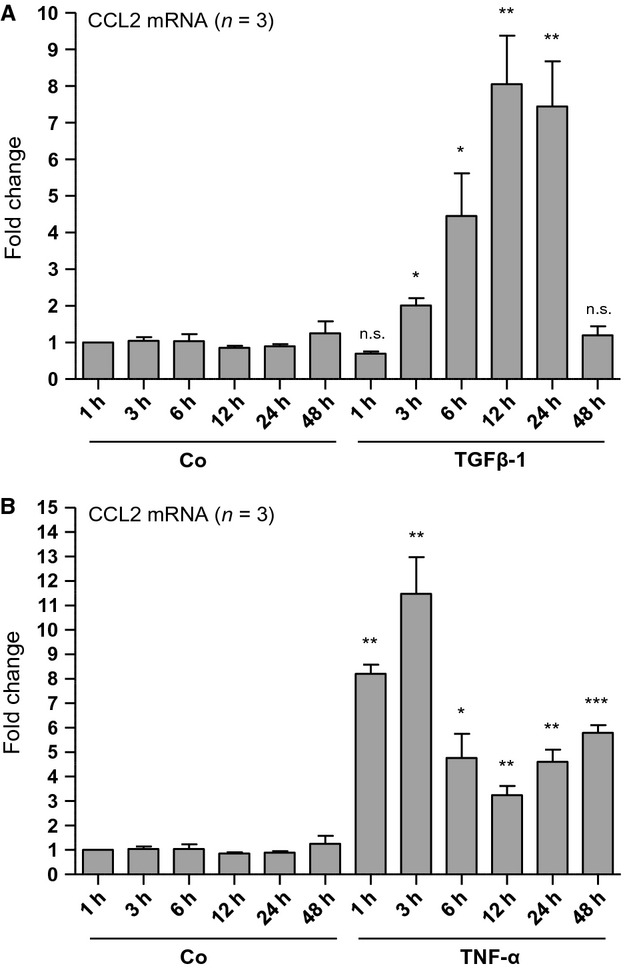
Time-dependent induction of CCL2 mRNA expression in TGF-*β*1- or TNF-*α*- stimulated human proximal tubular epithelial cell (PTC). HK-2 cells were serum- and supplement-starved for 48 h and subsequently stimulated with TGF-*β*1 (A) or TNF-*α* (B) (at a concentration of 10 ng/mL each) for the indicated periods of time. Real-time PCR analysis was performed as described in Methods. Data are given as fold induction above CCL2 mRNA control levels after normalizing to GAPDH mRNA expression. Each data point represents the average of three independent experiments with error bars corresponding to SEM (*P <* *0.05; **P <* *0.01; ***P <* *0.001).

**Figure 4:**
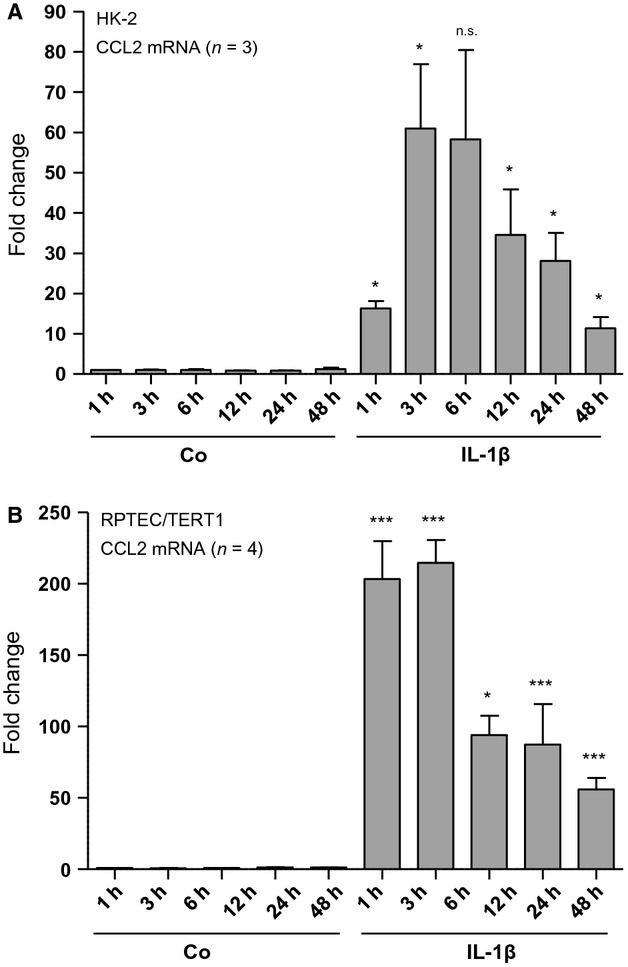
IL-1*β*-stimulated, time-dependent induction of CCL2 mRNA expression in two human proximal tubular epithelial cell (PTC) lines. (A) HK-2 cells were grown to a subconfluent state, serum- and supplement-starved for 48 h, and subsequently stimulated with 10 ng/mL IL-1*β* for the indicated periods of time. (B) RPTEC/TERT1 cells were grown to a confluent state, serum- and supplement-starved for 48 h, and subsequently stimulated with 10 ng/mL IL-1*β* for the indicated periods of time. Real-time PCR analysis was performed as described in Methods. Data are given as fold induction above CCL2 mRNA control levels after normalizing to GAPDH mRNA expression. Each data point represents the average of 3 (A) or 4 (B) independent experiments with error bars corresponding to SEM (*P <* *0.05; ***P <* *0.001).

Renal inflammation is induced as a protective response to a wide range of injuries in an attempt to eliminate the cause and promote repair, but ongoing inflammation promotes progressive renal fibrosis (Meng et al. 2014). Chemokines such as CCL2 and their receptors are key mediators involved in inflammatory cell interactions and recruitment, cellular adhesion, differentiation, and tissue damage in the setting of diabetic nephropathy (Navarro-González et al. 2011; Kanasaki et al. 2013; Wada and Makino 2013). Renal proximal tubular cells (PTC) represent important proinflammatory/profibrotic effector cells for OSM and several other proinflammatory/profibrotic ligands in these processes (Nightingale et al. 2004; Pollack et al. 2007; Elbjeirami et al. 2010; Sarközi et al. 2011, 2012; Tang and Lai 2012). Thus, we next studied mRNA expression of the chemokine CCL2 in human PTC selectively stimulated with IL-1*β*, TNF-*α,* or TGF-*β*1. As depicted in the following figures, all of these three ligands led to a strong time-dependent upregulation of CCL2 mRNA levels in HK-2 cells. TGF-*β*1-stimulated CCL2 mRNA expression started 3 h after ligand administration, was highest after 12 h (8.1-fold; *n* = 3; *P* < 0.01) and lasted for 24 h (Fig.3A). In contrast, TNF-*α*-induced CCL2 mRNA expression already after 1 h of stimulation, which reached a maximum after 3 h (11.5-fold; *n* = 3; *P* < 0.01) and lasted for at least 48 h (Fig.3B). Strongest CCL2 mRNA induction, however, occurred after 3 h of stimulation with IL-1*β* in both human PTC lines HK-2 (60.9-fold; *n* = 3; *P* < 0.05) and RPTEC/TERT1 (214.6-fold; *n* = 4; *P* < 0.001) (Fig.4). This rapid, potent, and long-lasting stimulation of CCL2 mRNA expression suggests that IL-1*β* indeed represents one of the strongest stimulators for CCL2 in human PTC.

**Figure 5:**
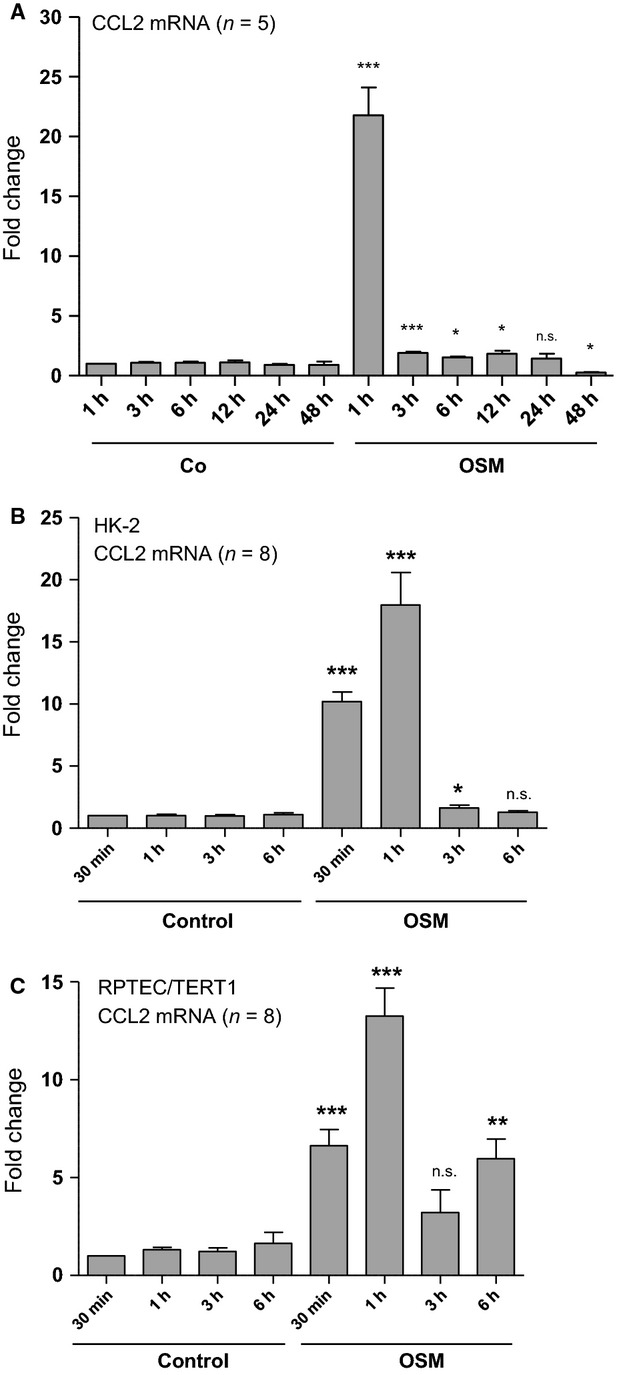
Time-dependent effects of oncostatin M (OSM) on CCL2 mRNA expression in human proximal tubular epithelial cell (PTC). (A, B) HK-2 cells were serum- and supplement-starved for 48 h, and subsequently stimulated with 10 ng/mL OSM for the indicated periods of time. (C) RPTEC/TERT1 cells were grown to a confluent state, serum- and supplement-starved for 48 h, and subsequently stimulated with 10 ng/mL OSM for the indicated periods of time. Real-time PCR analysis was performed as described in Methods. Data are given as fold induction above CCL2 mRNA control levels after normalizing to GAPDH mRNA expression. Each data point represents the average of 5 (A) or 8 (B, C) independent experiments with error bars corresponding to SEM (*P <* *0.05; **P <* *0.01; ***P <* *0.001).

A surprising and interesting result was obtained following analysis of time-dependent CCL2 mRNA expression in the presence of OSM (Fig.5). When compared with unstimulated control cells, OSM led to a fast and strong upregulation of CCL2 mRNA expression, which was 21.8-fold after 1 h. Subsequently CCL2 mRNA expression rapidly returned toward basal levels in between 3 and 24 h before this stimulation switched into a significant OSM-mediated 70% inhibition of basal CCL2 mRNA expression after 48 h of incubation (Fig.5A). An additional comparative study of OSM-mediated CCL2 mRNA expression between 30 min and 6 h in HK-2 (Fig.5B) and RPTEC/TERT1 (Fig.5C) cells revealed that OSM-induced CCL2 mRNA expression started in both human PTC lines as early as 30 min after ligand administration. Together these results confirm a rapid and sufficient OSM-mediated stimulation of CCL2 mRNA expression in two independent proximal tubular cell lines and suggest that OSM on its own may have an inhibitory effect on CCL2 mRNA expression in these cells at later time points.

### OSM has a strong additive stimulatory effect on CCL2 mRNA expression in the presence of IL-1β, TNF-α, and TGF-β1 for up to 24 h

**Figure 6:**
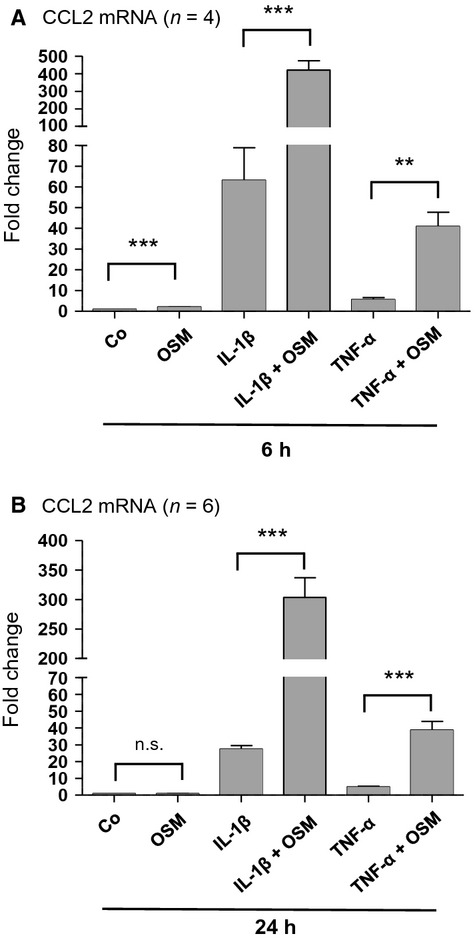
Oncostatin M (OSM)-mediated additive effect on CCL2 mRNA expression in IL-1*β*- and TNF-*α*-stimulated human proximal tubular epithelial cell (PTC). HK-2 cells were serum- and supplement-starved for 48 h, and subsequently stimulated for 6 h (A) or 24 h (B) with OSM alone, IL-1*β* alone, TNF-*α* alone (10 ng/mL each), with IL-1*β* in the presence of OSM (IL-1*β* + OSM) or with TNF-*α* in the presence of OSM (TNF-*α* + OSM) when compared with unstimulated controls. Real-time PCR analysis was performed as described in Methods. Data are given as fold induction above CCL2 mRNA control levels after normalizing to GAPDH mRNA expression. Each data point represents the average of 4 (A) or 6 (B) independent experiments with error bars corresponding to SEM (**P <* *0.01; ***P <* *0.001).

In order to get a better understanding of OSM's potential proinflammatory effects in human PTC we next studied its effects on CCL2 mRNA expression in the presence of IL-1*β*, TNF-*α*, and TGF-*β*1 (all were administered at a concentration of 10 ng/mL). After 6 h of incubation, OSM on its own stimulated CCL2 mRNA expression by 2.1-fold (*n* = 4; *P* < 0.001) in HK-2 cells (Fig.6A). Administration of IL-1*β* in the presence of OSM for 6 h led to a 6.7-fold additive stimulatory effect of the cytokine on CCL2 mRNA expression when compared with IL-1*β* alone (from 63.3- to 420.8-fold; *n* = 4; *P* < 0.001) (Fig.6A). This additive effect of a combined treatment with IL-1*β* and OSM was further increased and reached a value of 11.0-fold after 24 h (from 27.5- to 303.6-fold; *n* = 6; *P* < 0.001) albeit OSM on its own did not affect basal CCL2 mRNA levels at this time point (Fig.6B). Moreover, we observed a similar synergistic induction of CCL2 mRNA expression upon treatment of the cells with OSM in combination with either TNF-*α* (Fig.6) or TGF-*β*1 (data not shown). Stimulation of HK-2 cells with TNF-*α* and OSM led to 7.3- and 7.8-fold additive effects after 6 and 24 h, respectively, when compared with TNF-*α* alone (*n* = 4; *P* < 0.01 and *n* = 6; *P* < 0.001) (Fig.6). When a combination of TGF-*β*1 and OSM was administered, CCL2 mRNA expression was 3.7- and 4.0-fold higher after 6 and 24 h, respectively, when compared with TGF-*β*1 treatment alone (*n* = 4; *P* < 0.01 and *n* = 6; *P* < 0.001) (data not shown). These results suggest that OSM exerts a strong additive stimulatory effect on CCL2 mRNA expression in the presence of either one of these three ligands for up to 24 h.

### OSM's additive stimulatory effect on IL-1β-induced CCL2 mRNA expression in HK-2 cells is partially mediated by STAT3

**Figure 7:**
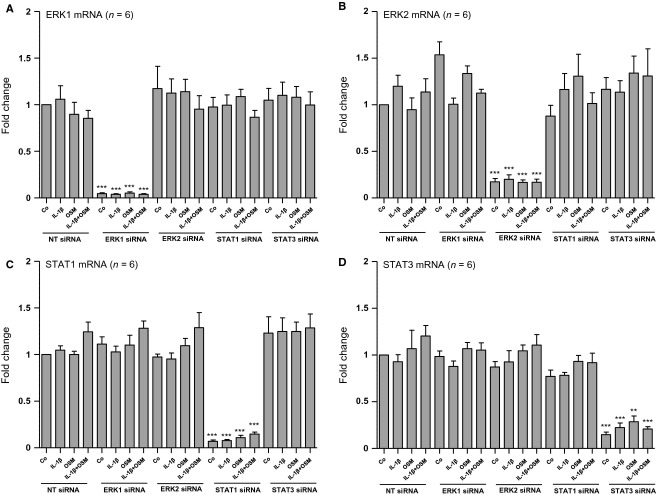
siRNA-mediated selective silencing of ERK1, ERK2, STAT1 and STAT3 in HK-2 cells. Serum starved cells were transfected with nontargeting siRNA (NT siRNA), ERK1 siRNA, ERK2 siRNA, STAT1 siRNA or STAT3 siRNA for 72 h, followed by stimulation for 1 h with 10 ng/mL IL-1*β* alone, oncostatin M (OSM) alone or with the combination of the two cytokines (IL-1*β* + OSM). The efficiency and selectivity of siRNA treatments was assessed by real-time PCR analysis. (A) Real-time PCR analysis of ERK1 mRNA expression in cells exposed to ERK1 siRNA when compared with ERK2 siRNA, STAT1 siRNA, STAT3 siRNA and NT siRNA. (B) Real-time PCR analysis of ERK2 mRNA expression in cells exposed to ERK2 siRNA when compared with ERK1 siRNA, STAT1 siRNA, STAT3 siRNA and NT siRNA. (C) Real-time PCR analysis of STAT1 mRNA expression in cells exposed to STAT1 siRNA when compared with ERK1 siRNA, ERK2 siRNA, STAT3 siRNA and NT siRNA. (D) Real-time PCR analysis of STAT3 mRNA expression in cells exposed to STAT3 siRNA when compared with ERK1 siRNA, ERK2 siRNA, STAT1 siRNA and NT siRNA. Each data point represents the average of six independent experiments with error bars corresponding to SEM (**P <* *0.01 or ***P <* *0.001 vs. NT siRNA-treated controls).

**Figure 8:**
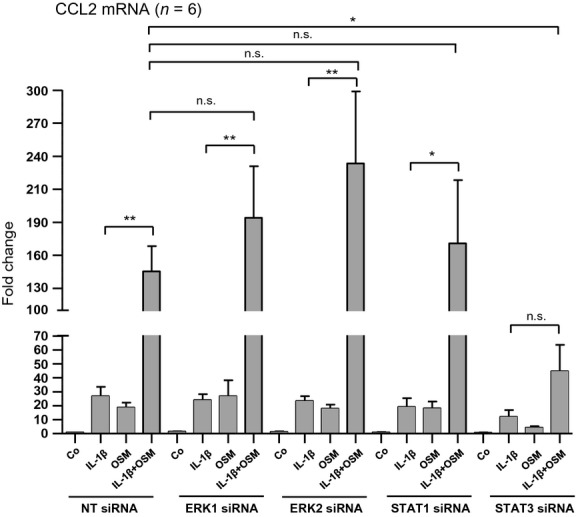
Silencing of STAT3 attenuates the additive stimulatory effect of oncostatin M (OSM) on CCL2 mRNA expression in the presence of IL-1*β*. HK-2 cells were transfected with nontargeting siRNA (NT siRNA), ERK1 siRNA, ERK2 siRNA, STAT1 siRNA or STAT3 siRNA. Seventy-two hours posttransfection cells were treated with 10 ng/mL IL-1*β* alone, 10 ng/mL OSM alone or with a combination of the two cytokines (IL-1*β* + OSM) for 1 h. The efficiency and selectivity of siRNA treatments and their effect on CCL2 mRNA expression was assessed by real-time PCR analysis. Each data point represents the average of six independent experiments with error bars corresponding to SEM (*P <* *0.05; **P <* *0.01).

We have recently published that OSM's inhibitory effect on TGF-*β*1-induced CTGF mRNA expression is mainly driven by STAT3 (Sarközi et al. 2012). To find out which signaling mechanisms are involved in OSM's early stimulatory effect on CCL2 mRNA expression as well as in its additive effect on IL-1*β*-induced CCL2 mRNA expression, we performed siRNA-mediated selective knockdown of distinct intracellular signaling molecules. Successful selective silencing of the signaling molecules ERK1, ERK2, STAT1, and STAT3 in HK-2 cells is depicted in Figure7. The effects of these specific interventions on CCL2 expression in the absence and presence of IL-1*β* and/or OSM are shown in Figure8. Selective silencing of either ERK1 or ERK2 revealed a tendency toward an increased additive effect of OSM on IL-1*β*-stimulated CCL2 mRNA expression after 1 h of ligand administration, which did not reach significance when compared with control cells transfected with nontargeting siRNA (NT siRNA). In contrast, siRNA-mediated silencing of STAT3 reduced OSM's synergistic effect on IL-1*β*-stimulated CCL2 mRNA expression after 1 h by 25.4% (Fig.8). In control cells transfected with nontargeting siRNA (NT siRNA) IL-1*β*-stimulated CCL2 mRNA expression reached a value of 26.9-fold in the absence of OSM and 135.5-fold in the presence of OSM (*P* < 0.01; *n* = 6), while in cells transfected with STAT3 siRNA IL-1*β*-driven CCL2 mRNA induction was 11.9- and 44.8-fold in the absence and in the presence of OSM, respectively (*P* = 0.12; *n* = 6). Amplification of CCL2 mRNA expression induced by combined administration of IL-1*β* and OSM when compared with IL-1*β* stimulation alone was still highly significant in those cells in which STAT1 was silenced (Fig.8). These results suggest that OSM's additive stimulatory effect on IL-1*β*-induced CCL2 mRNA expression is at least partially mediated by STAT3 but neither by STAT1 nor by ERK1 or ERK2.

### p65 NFκB/RelA and SMAD2/3 differentially affect CCL2 expression in proximal tubular HK-2 cells stimulated by a combination of IL-1β and OSM

**Figure 9:**
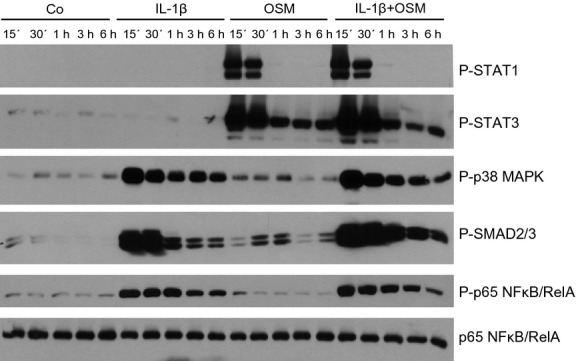
Time-dependent effects of IL-1*β* and oncostatin M (OSM) on phosphorylation of STAT1, STAT3, p65 NF*κ*B/RelA, p38 MAPK and SMAD2/3 in human proximal tubular epithelial cell (PTC). Serum starved HK-2 cells were stimulated with IL-1*β*, OSM or a combination of the two cytokines (10 ng/mL each) for the indicated periods of time. Protein expression of p65 NF-*κ*B/RelA shows equal protein loading. One representative Western blot of three separate experiments is depicted.

The fact that OSM-stimulated STAT3 seems to be responsible for only 25% of the synergistic CCL2 mRNA induction in the presence of both IL-1*β* and OSM raises the question, which other signaling pathways might be involved. Thus, we next investigated several signaling molecules, which are likely to be activated by IL-1*β* and/or OSM in HK-2 cells. As depicted in Figure9, OSM but not IL-1*β* rapidly stimulated phosphorylation of both STAT1 and STAT3. While OSM-mediated STAT1 phosphorylation was reduced to basal levels after 1 h, OSM-mediated STAT3 phosphorylation remained elevated for at least 6 h. No additive effect on STAT1/3 phosphorylation was detectable in the presence of both IL-1*β* and OSM. Interestingly, IL-1*β* led to a rapid and long-lasting phosphorylation of p38 MAPK, SMAD2/3, and p65 NF*κ*B/RelA (Fig.9). While OSM alone only slightly stimulated SMAD2/3 phosphorylation after 30 min and 1 h, administration of both IL-1*β* and OSM revealed a long-lasting additive effect on SMAD2/3 phosphorylation (Fig.6).

**Figure 10:**
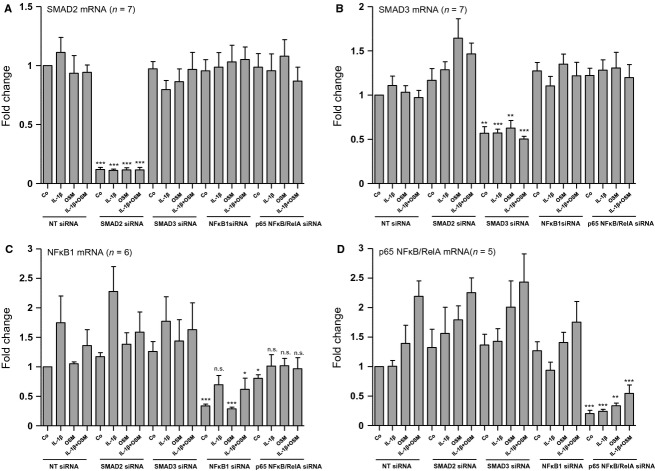
siRNA-mediated selective silencing of SMAD2, SMAD3, NF*κ*B1 and p65 NF*κ*B/RelA in HK-2 cells. Serum starved cells were transfected with nontargeting siRNA (NT siRNA), SMAD2 siRNA, SMAD3 siRNA, NF*κ*B1 siRNA or p65 NF*κ*B/RelA siRNA for 72 h, followed by stimulation for 1 h with 10 ng/mL IL-1*β* alone, oncostatin M (OSM) alone or with the combination of the two cytokines (IL-1*β* + OSM). The efficiency and selectivity of siRNA treatments was assessed by real-time PCR analysis. (A) Real-time PCR analysis of SMAD2 mRNA expression in cells exposed to SMAD2 siRNA when compared with SMAD3 siRNA, NF*κ*B1 siRNA, p65 NF*κ*B/RelA siRNA and NT siRNA. (B) Real-time PCR analysis of SMAD3 mRNA expression in cells exposed to SMAD3 siRNA when compared with SMAD2 siRNA, NF*κ*B1 siRNA, p65 NF*κ*B/RelA siRNA and NT siRNA. (C) Real-time PCR analysis of NF*κ*B1 mRNA expression in cells exposed to NF*κ*B1 siRNA when compared with SMAD2 siRNA, SMAD3 siRNA, p65 NF*κ*B/RelA siRNA and NT siRNA. (D) Real-time PCR analysis of p65 NF*κ*B/RelA mRNA expression in cells exposed to p65 NF*κ*B/RelA siRNA when compared with SMAD2 siRNA, SMAD3 siRNA, NF*κ*B1 siRNA and NT siRNA. Each data point represents the average of 7 (A, B), 6 (C) or 5 (D) independent experiments with error bars corresponding to SEM (*P <* *0.05; **P <* *0.01; ***P <* *0.001 vs. NT siRNA-treated controls).

**Figure 11:**
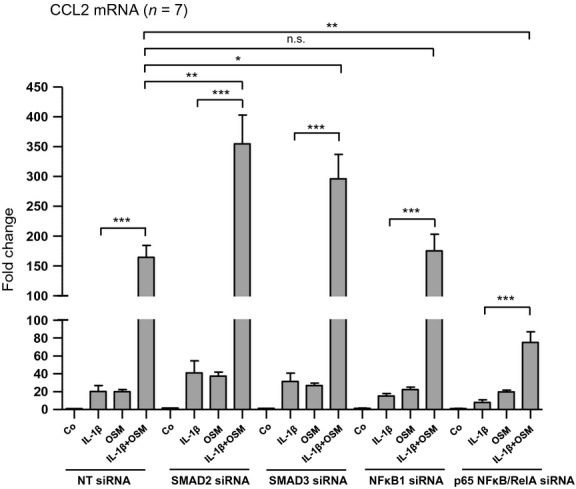
Differential effects of SMAD2, SMAD3, NF*κ*B1 and p65 NF*κ*B/RelA silencing on CCL2 mRNA expression in HK-2 cells. HK-2 cells were transfected with nontargeting siRNA (NT siRNA), SMAD2 siRNA, SMAD3 siRNA, NF*κ*B1 siRNA or p65 NF*κ*B/RelA siRNA. Seventy-two hours posttransfection cells were treated with 10 ng/mL IL-1*β* alone, 10 ng/mL oncostatin M (OSM) alone or with a combination of the two cytokines (IL-1*β* + OSM) for 1 h. The efficiency and selectivity of siRNA treatments and their effect on CCL2 mRNA expression was assessed by real-time PCR analysis. Each data point represents the average of seven independent experiments with error bars corresponding to SEM (*P <* *0.05; **P <* *0.01; ***P <* *0.001).

In order to investigate their role in the regulation of CCL2 mRNA expression we next performed siRNA-mediated selective knockdown of SMAD2, SMAD3, NF*κ*B1 or p65 NF*κ*B/RelA (Fig.10). When compared with nontargeting siRNA-treated cells, downregulation of either SMAD2 or SMAD3 led to an additional increase in CCL2 mRNA expression when the cells were stimulated with both IL-1*β* and OSM (2.2-fold in the case of SMAD2; *n* = 7; *P* < 0.01 and 1.8-fold in the case of SMAD3; *n* = 7; *P* < 0.05) (Fig.11). These results suggest that neither SMAD2 nor SMAD3 are involved in the additive stimulatory effect induced by coadministration of IL-1*β* and OSM but rather attenuate CCL2 mRNA expression. In contrast, exposure of the cells to p65 NF*κ*B/RelA siRNA revealed a tendency to reduce CCL2 mRNA expression, which was highly significant in the presence of both IL-1*β* and OSM (*P* < 0.01; *n* = 7) when compared with nontargeting siRNA-treated cells (Fig.11). However, the stimulatory effect of IL-1*β* and OSM coadministration when compared with IL-1*β* alone reached similar values after p65 NF*κ*B/RelA silencing as we observed in control samples (8.1-fold in NT siRNA-treated cells and 9.5-fold in p65 NF*κ*B/RelA siRNA-treated cells; *P* < 0.001 each; *n* = 7). Moreover, silencing of NF*κ*B1 did not significantly affect CCL2 mRNA expression under any one of the conditions tested. All together these results suggest that p65 NF*κ*B/RelA is at least partially responsible for the IL-1*β*-mediated induction of CCL2 mRNA, both in the absence and in the presence of OSM.

## Discussion

OSM represents a cytokine that has been discussed in the context of inflammatory kidney disease and tubulointerstitial fibrogenesis as well as a mediator of renal tissue remodeling and regeneration (Nightingale et al. 2004; Pollack et al. 2007; Liu 2010; Sarközi et al. 2011, 2012). We have previously reported evidence that OSM may have a dual role in the regulation of proximal tubular epithelial cell (PTC) differentiation and phenotype (Pollack et al. 2007; Sarközi et al. 2011, 2012). Besides triggering morphological alterations indicative of renal tubular epithelial-mesenchymal transition (EMT) (Pollack et al. 2007), OSM can also act as an inhibitor of TGF-*β*1-induced matricellular protein expression (Sarközi et al. 2011, 2012), which suggests that this cytokine may have both pro- and antifibrotic effects. Here, we report novel proinflammatory actions of OSM in the absence and in the presence of IL-1*β* and TNF-*α*. Three major findings can be derived from the present study: (1) Although OSM inhibits the expression of matricellular proteins TSP-1 and TNC, which have been stimulated by proinflammatory cytokines IL-1*β* or TNF-*α*, it acts synergistically with these two ligands on inducing CCL2/MCP-1 mRNA expression for up to 24 h. (2) In contrast to OSM, which stimulates both STAT1/3 and ERK1/2 signaling (Pollack et al. 2007; Sarközi et al. 2011), IL-1*β* is a potent activator of p65 NF*κ*B/RelA, SMAD2/3, and p38 MAPK in proximal tubular HK-2 cells. (3) While p65 NF*κ*B/RelA is involved in IL-1*β*-mediated stimulation of CCL2 mRNA, superinduction of CCL2 mRNA expression in the presence of both OSM and IL-1*β* at least partially depends on STAT3 signaling.

We and others have previously reported evidence that OSM is able to stimulate cellular events, which are associated with dedifferentiation of human PTC and which are comparable to a mechanism known as epithelial-mesenchymal transition (EMT) (Nightingale et al. 2004; Pollack et al. 2007). However, we have also demonstrated, that OSM can diminish TGF-*β*1-induced expression of the transcriptional EMT mediator FoxC2 and attenuates basal as well as TGF-*β*1-induced expression of matricellular proteins CTGF, TSP-1, TNC, and SPARC, which suggests that this IL-6 family cytokine may have a unique role in the regulation of cellular mechanisms associated with renal tubular fibrogenesis (Sarközi et al. 2011). In the present paper we show that OSM is able to inhibit the expression of matricellular proteins such as TSP-1 and TNC even after their stimulation with proinflammatory cytokines such as IL-1*β* and TNF-*α*. More importantly, however, is the ability of OSM to act as a short-term stimulator of CCL2 mRNA. In addition, OSM massively enhances CCL2 mRNA expression for up to 24 h when administered together with IL-1*β* or TNF-*α*. These results are in line with the finding that OSM alone, or in synergy with IL-1*β*, or together with TNF-*α*, potently induces expression of the proinflammatory cytokine IL-6 in isolated nonarthritic, wild-type mouse synovial fibroblasts (Le Goff et al. 2014). Time-course experiments of OSM-mediated CCL2 mRNA expression revealed, that OSM on its own leads to a rapid and potent CCL2 mRNA upregulation within the first hour of stimulation, which is subsequently reduced from 21.8-fold after 1 h of stimulation to a value of about 2.0-fold after 3 h, and which remains significantly elevated at this level for at least 12 h. After 24 h of treatment, however, OSM-mediated CCL2 mRNA expression returns to basal values before OSM turns to inhibit this gene. These results suggest that OSM exerts time-dependent differential effects during proximal tubular inflammation. While early after injury (1–24 h) OSM has synergistic effects with IL-1*β* or TNF-*α* with respect to CCL2 expression, it may exert inhibitory actions on both profibrotic (2–24 h) and proinflammatory genes (after 48 h) at later time points. Thus, with respect to matricellular protein expression, OSM is likely to attenuate excessive matrix production and/or deposition and, finally, fibrogenesis (Sarközi et al. 2011, 2012). In the case of proinflammatory CCL2 expression, it may stimulate acute inflammation via its synergistic effect with other proinflammatory cytokines as a protective effect early after injury, but may attenuate chronic inflammation at later time points (Meng et al. 2014). Absence of long-term inhibitory OSM action, on the other hand, would promote progressive renal injury and fibrosis under these circumstances.

To get insight into the molecular mechanism of CCL2 mRNA expression and its regulation in human proximal tubular HK-2 cells in the presence and absence of IL-1*β* and OSM, we investigated several signaling molecules, which are likely to be activated by these two cytokines. Our present results do not only confirm that OSM is a potent activator of STAT1 and STAT3 signaling (Pollack et al. 2007; Sarközi et al. 2011, 2012) in these cells but also provides novel evidence that IL-1*β* transduces its signals via p38 MAPK, SMAD2/3, and NF*κ*B cascades. OSM but not IL-1*β* induced a strong and transient phosphorylation of STAT1, which returned to basal levels as early as 1 h after stimulation. In contrast, OSM-mediated STAT3 activation was more sustained and lasted for at least for 6 h. Thus, it is conceivable that silencing of STAT3 but not of STAT1 reduced CCL2 mRNA expression stimulated by OSM alone or by OSM in the presence of IL-1*β*. When compared with cells transfected with nontargeting siRNA, silencing of STAT3 resulted in an approximate 25% reduction in OSM's synergistic effect on CCL2 mRNA expression in the presence of IL-1*β*. This fact suggests that additional signaling molecules are likely to be involved in the superinduction of CCL2 mRNA in the presence of both OSM and IL-1*β*. Thus, we also performed knockdown experiments of other signaling molecules, which are known to be involved in signal transduction mediated by OSM and/or IL-1*β*. Of these, p65 NF*κ*B/RelA silencing revealed a tendency to reduce CCL2 mRNA expression, which was highly significant in the presence of both IL-1*β* and OSM when compared with nontargeting siRNA-treated cells suggesting, that p65 NF*κ*B/RelA is at least partially responsible for the IL-1*β*-mediated induction of CCL2 mRNA, both in the absence and in the presence of OSM.

All together it is tempting to speculate that OSM in the presence of IL-1*β* may exert early synergistic effects on the expression of proinflammatory CCL2 via STAT3 and p65 NF*κ*B/RelA, respectively, while inhibiting this gene at later time points, for example after 48 h. This mechanism would fit to OSM's long-lasting inhibitory effects on matricellular protein expression, which may attenuate excessive matrix deposition and, thus, renal fibrogenesis.
